# Exploration of the mechanism of Taohong Siwu Decoction for the treatment of ischemic stroke based on CCL2/CCR2 axis

**DOI:** 10.3389/fphar.2024.1428572

**Published:** 2024-08-29

**Authors:** Jingjing Li, Lijuan Zhang, Sujun Xue, Chao Yu, Yumeng Li, Shuangping Li, Qingping Ye, Xianchun Duan, Daiyin Peng

**Affiliations:** ^1^ Department of Pharmacy, The First Affiliated Hospital of Anhui University of Chinese Medicine, Hefei, China; ^2^ School of Pharmacy, Anhui University of Chinese Medicine, Hefei, China; ^3^ Key Laboratory of Chinese Medicinal Formula Research, Anhui University of Chinese Medicine, Hefei, China

**Keywords:** Taohong Siwu Decoction (THSWD), ischemic stroke, bioinformatics, CCL2/CCR2 axis, immune inflammation

## Abstract

**Background and aims:**

Taohong Siwu Decoction (THSWD) is a traditional Chinese herbal prescription that is effective for ischemic stroke, Whether THSWD regulates the CCL2/CCR2 axis and thus reduces the inflammatory response induced by ischemic stroke is not known. The aim of this study was to elucidate the mechanism of action of THSWD in the treatment of ischemic stroke using bioinformatics combined with *in vitro* and *in vivo* experiments.

**Methods:**

R language was used to analyze middle cerebral artery occlusion/reperfusion (MCAO/R) rat transcriptome data and to identify differential gene expression following THSWD treatment. Gene set enrichment analysis (GSEA) was used to analyze the gene set enrichment pathway of MCAO/R rats treated with THSWD. PPI networks screened key targets. The Human Brain Microvascular Endothelial Cells (HBMEC) Oxygen Glucose Deprivation/Reoxygenation (OGD/R) model and SD rat models of MCAO/R were established. FITC-dextran, immunofluorescence, flow cytometry, ELISA, immunohistochemistry, Western blotting, and RT-qPCR were performed to identify potential treatment targets.

**Results:**

A total of 515 differentially expressed genes of THSWD in MCAO/R rats were screened and 92 differentially expressed genes of THSWD potentially involved in stroke intervention were identified, including Cd68, Ccl2, and other key genes. *In vitro*, THSWD reversed the increase in permeability of HBMEC cells and M1/M2 polarization of macrophages induced by CCL2/CCR2 axis agonists. *In vivo*, THSWD improved nerve function injury and blood-brain barrier injury in MCAO/R rats. Further, THSWD inhibited the infiltration and polarization of macrophages, reduced the expression of IL-6, TNF-α, and MMP-9, and increased the expression of IL-4, while reducing the gene and protein expression of CCL2 and CCR2.

**Conclusion:**

THSWD may play a protective role in ischemic stroke by inhibiting the CCL2/CCR2 axis, reducing the infiltration of macrophages, and promoting the polarization of M2 macrophages, thereby reducing inflammatory damage, and protecting injury to the blood-brain barrier.

## 1 Introduction

The burden of ischemic stroke on the lives of individuals is increasing worldwide, and the situation is particularly serious in China, which is reported to account for nearly one-third of the global total of stroke-related deaths (Wu et al., 2022). Ischemic stroke is characterized by an abrupt interruption of blood flow to a particular brain region of the brain, leading to inadequate oxygen and glucose provided to the ischemic tissue. Timely blood reperfusion can save dying tissue but may also lead to secondary damage to infarct tissue and to the blood-brain barrier (BBB) (Xie et al., 2022). Therefore, it is meaningful to investigate the mechanism of ischemic stroke and its treatment.

CCL2, or monocyte chemoattractant protein-1 (MCP-1), was the first human monocyte chemokine to be discovered, and its biological function is mediated by binding to its cognate receptor, the CC motif chemokine receptor 2 (CCR2) (Bianconi et al., 2018). CCL2 binds to CCR2 to mediate monocyte migration into the brain (Bai et al., 2023a). Monocytes differentiate into macrophages or dendritic cells (DC) according to the local tissue environment. M1 macrophages are pro-inflammatory at the site of brain injury, producing cytokines such as interleukin (IL)-6 and tumor necrosis factor (TNF)-α (Bai et al., 2023b). The BBB is connected by tight junction proteins on the surface of endothelial cells, and BBB damage is related to the influx of inflammatory cells (Salvador et al., 2015). Early research has shown that CCL2 in various pathological conditions mediated by BBB damage (Errede et al., 2022), with the increasing of BBB permeability, infiltrating chemical mediators and inflammatory cells (such as macrophages) cause inflammation, which further enhances BBB permeability. Additionally, after ischemic stroke, functional damage to neurons is aggravated, resulting in neuronal apoptosis, during which a large number of microglia/macrophages are activated, which, in turn, produces pro-inflammatory cytokines, further aggravating neuronal apoptosis during ischemic stroke (Shao et al., 2022). Inhibiting macrophage infiltration and maintaining BBB integrity have been confirmed in studies to be mutually reinforcing (Cao et al., 2021). Therefore, down-regulating chemokine expression and preventing infiltration of inflammatory cells (such as macrophages) is likely to be an effective way to reduce BBB permeability and protect the brain from injury (Payen et al., 2003).

Taohong Siwu Decoction (THSWD) is composed of Tao-ren, Hong-hua, Dang-gui, Chuan-xiong, and Bai-shao in certain proportions. This prescription first appeared in “Yi Zong Jin Jian” written by Wu Qian during the Qing Dynasty and is a classic formula for activating blood circulation and removing blood stasis. Clinically, it has been used for the treatment and prevention of cerebrovascular diseases (Duan et al., 2018). Previous studies have shown that THSWD is neuroprotective in brain injury models and can reduce ischemic stroke injury by inhibiting cell necrosis, neuroinflammation, and cell pyrodeath (Wang et al., 2020; Wang et al., 2021); THSWD can also treat ischemic stroke by improving vascular structure and promoting angiogenesis (Tang et al., 2024). However, few studies have evaluated the transcriptomics of THSWD therapy for ischemic stroke based on bioinformatic methods. In this study, we investigated the immunoinflammatory-related mechanisms of THSWD for ischemic stroke based on bioinformatics methods and performed *in vitro* and *in vivo* experiments to validate and investigate the role played by CCL2/CCR2 in the therapeutic process of THSWD for ischemic stroke. To complement the pharmacological effects of THSWD in the treatment of ischemic stroke. Nimodipine has neuroprotective effects against ischemic stroke and has been proven to reduce the area of brain tissue damage after cerebral infarction, as well as improve neurological functional impairment following cerebral ischemia (Sun et al., 2023). Therefore, in this study, we used nimodipine as a positive control.

## 2 Materials and methods

### 2.1 Medicinal materials and reagents

Tao-ren (NO: 20230818), Hong-hua (NO: 2307273), Dang-gui (NO: 2230301), Chuan-xiong (NO: 2307091), Bai-shao (NO: 2307200022), and Shu-di (NO: 2307173) medicinal materials (ratio: 3:2:4:3:3:2) were all purchased from the First Affiliated Hospital of Anhui University of Chinese Medicine. For the preparation of THSWD water extract, the herbs were prepared according to the THSWD ratios, extracted with water, and then concentrated using a rotary evaporator to a concentrate with a drug content of 1.8 g/mL.

Nimodipine (cat.no. 210507) was purchased from Yabo Pharmaceutical Group Co., LTD., TRIzol extraction reagent (cat.no. 15596026) was purchased from Thermo Fisher Scientific, mirVana™ miRNA isolation kit (cat.no. AM1561) from Ambion, CCK8 test kit (cat.no. CR2112050) from Servicebio, anti-Human CD86 (B7-2) (cat.no. A10944), anti-Human/Mouse CD11b (cat.no. A11042), were both purchased from MultiSciences, APC anti-Human CD206 (LW1218) was purchased from Elabscience, human TNF-α (July 2023), Human matrix metalloproteinase 9 (MMP-9) (July 2023), human IL-6 (July 2023), human IL-4 (July 2023), rat TNF-α (May 2023), rat MMP-9 (May 2023), rat IL-6 (May 2023), rat IL-4 (May 2023) were purchased from Quanzhou Ruixin Biotechnology Co., Ltd., claudin-5 (cat.no. 23o5501), CCL2 (cat.no. 60d7593), and CCR2 (cat.no. 60e2906) were purchased from Affinity, DAPI (cat.no. 22334227) from Beyotime, FITC-Dextran (cat.no. 178439) from MCE, β-actin (cat.no. 19AW0505), goat anti-mouse IgG (cat.no. 142637), and goat anti-rabbit IgG (cat.no. 139931) were purchased from Zs-BIO.

### 2.2 Animals and model preparation

Male SPF grade SD rats (150–180 g) were purchased from Liaoning Changsheng Biotechnology Co., Ltd. The animal experiments were approved by the Experimental Animal Ethics Committee of the Anhui University of Chinese Medicine (Permit Number: LLSC20160036).

Establishment of the MCAO/R model was described previously (Ji et al., 2022). Briefly, the ophthalmic scissors made a 1.0-cm incision in the middle of the neck. The proximal end of the right common carotid artery (CCA) and the external carotid artery (ECA) were ligated with a 5-cm suture. After closing the distal end of the internal carotid artery (ICA) with an artery clamp, a nylon wire was inserted into the intersection of ECA and ICA to an inward depth of (18.5 ± 0.5) mm, obstruction of the middle cerebral artery was thus achieved and the cord was removed after 2 h of ischemia, and the MCAO/R model was created. In the sham group, only ECA and ICA were ligated, and no other special treatment was performed.

### 2.3 Bioinformatics approaches to study potential immunoinflammation-related targets of THSWD for the treatment of ischemic stroke

#### 2.3.1 Differential expression gene screening and GSEA analysis

RNA sequencing data was obtained from on a preliminary high-throughput sequencing study (Duan et al., 2018), Using EdgeR in R 4.2.1 compared DEG expression in the MCAO/R and sham groups and between the THSWD and MCAO/R groups, respectively. *p* < 0.05 and |logFC| > 1 were defined as significant difference criteria. Venny 2.1.0 (https://bioinfogp.cnb.csic.es/tools/venny/) was used to screen DEGs [MCAO/R vs sham (MVSC) and THSWD vs MCAO/R (DVSM)].

Transcriptome data was collated and analyzed using gene set enrichment analysis (GSEA) and enrichplot with R packages (clusterProfiler, org. Rn.eg.dbde, Enrichplot, pathview, dplyr, reshape2, ggplot2, and ggridges), where pvalueCutoff = 0.05 were used for the analyses. When both the *P*-value and the FDR were <0.05, it indicated a significant gene set enrichment.

#### 2.3.2 Immunoinflammation-related gene screening and enrichment analysis

R4.2.1 was used to screen immunoinflammatory genes in the GO and KEGG databases. After dereprocessing, the two groups of immunoinflammatory genes were mixed with DVSM and MVSC to obtain immunoinflammatory-related differential genes for the THSWD intervention in ischemic stroke. R packages (ClusterProfiler, BiocManager, AnnotationHub, AnnotationDbi, and ggplot2) were applied to GO and KEGG analysis to identify immunoinflammatory-related differential gene expression following THSWD treatment of ischemic stroke with pvalueCutoff = 0.05 and qvalueCutoff = 0.05.

#### 2.3.3 Protein-protein interaction network construction and key gene screening

The protein-protein interaction (PPI) network map of genes related to immune inflammation was created using the String database (https://cn.string-db.org/) and imported into Cytoscape 3.9.1 for the visualization of the selected genes. The CytoHubba analysis module network was also used and the MCC algorithm was selected to identify genes with the highest score as key genes.

### 2.4 Immunoinflammatory protective effect of THSWD on the OGD/R model of human brain microvascular endothelial cells

#### 2.4.1 Preparation of THSWD drug-containing serum

Twenty-four healthy SD rats, 10 rats were grafted with THSWD (18 g/kg) once a day in the morning and once a night for 3 consecutive days. The remaining rats received an equal amount of saline. One hour after the last gavage, 3% pentobarbital sodium was injected intraperitoneally, blood was taken from the abdominal aorta, left for rest, serum was taken after centrifugation, bacteria were removed with a 0.22 μm filter, and then stored at −80°C after subpacking.

#### 2.4.2 Grouping and intervention methods

Human Brain Microvascular Endothelial Cells (HBMEC) were randomly divided into 6 groups: control, OGD/R, OGD/R + CCL2/CCR2 axis agonist (Agonist), OGD/R + CCL2/CCR2 axis inhibitor (Inhibitor), OGD/R + THSWD (THSWD), OGD/R + CCL2/CCR2 axis agonist + THSWD (THSWD + Agonist) groups. Molding method: HBMEC cells were seeded in the lower chamber of the 6-well plate and THP-1 cells were seeded in the coculture chamber. After 48 h of coculture with PMA100 ng/mL, HBMEC cells, except for the control group, were rinsed with PBS, then glucose-free DMEM medium was added to the anoxic incubator for 2 h, and the medium was changed into a complete medium and returned to the original incubator for 24 h. For the Agonist group: the final concentration of CCL2 was 200 ng/mL and treated duration was 24 h. For the Inhibitor group, the CCR2 inhibitor RS102896 was added to a final concentration 10 μM and cells were treated for 24 h. For the THSWD group, 10% drug-containing serum was added and cells were treated for 48 h. For the THSWD + Agonist group, drug-containing serum was added first, and agonist was added 24 h later and treatment continued for 24 h. For the control group and the OGD/R group there was no other processing.

#### 2.4.3 Cell viability was detected by CCK-8

According to the manufacturer’s instructions for the CCK-8 (CellCountingKit-8) test kit, cells were inoculated in 96-well culture plates at a density of 1×10^5^, with 100 μL per well and 6 multiple wells per set. The inoculated cell culture plates were placed in the incubator overnight for culture, and then the cells were molded and dosed, 10 μL CCK8 was added to each well, and the culture was continued for 1 h. The light absorption values of each hole were measured at OD450 nm in the ELISA, and blank holes were set.

#### 2.4.4 FITC-glucan was used to measure endothelial cell permeability

HBMEC cells were inoculated in the upper inner compartment of the Transwell compartment and the lower compartment contained THP-1 cells. After cell growth and fusion, serum-free medium was cultured overnight to synchronize the cells. After the cells were molded and dosed, 1 mg/mL of FITC-Dextran was added and cells were placed in the incubator for 5 h. Samples of 100 μL were taken from the top chamber and the bottom chamber of each bilayer chamber, and the fluorescence intensity of the samples was detected by a fluorescence enzyme spectrometer. The excitation wavelength was 485 nm and the emission wavelength was 595 nm. The volume of fluid in the bottom chamber was also measured.

#### 2.4.5 Claudin-5 protein expression was determined by immunofluorescence

The cocultured cells were inoculated in the tablet at an appropriate density. After molding, the culture medium was discarded and cleaned with PBS 3 times and 4% paraformaldehyde was added to fix cells for 20 min, and then washed with PBS 3 times. A PBS solution containing 0.25%TritonX-100 was added and incubated for 10 min. PBS was cleaned three times. Next, a 4% BSA solution was added to seal cells for 30 min. The BSA solution was then removed and the primary antibody was added, incubated at 4°C overnight, washed with PBS, before the secondary antibody was added, and incubated for 3 h and washed 3 times with PBS. The slides were sealed with antifluorescence quenched tablets containing DAPI and observed and photographed by a high-resolution fluorescence microscope.

#### 2.4.6 Macrophage polarization was detected by flow cytometry

The treated cells were collected, mixed with anti-CD11 and CD86 antibodies, and incubated at room temperature for 15 min away from light. The cells were permeated according to instructions, the membrane was broken, the anti-CD206 antibody was added and incubated at room temperature for 15 min away from light. After washing, cells were resuspended with an appropriate amount of PBS containing 5% FBS and then filtered by 200 mesh.

#### 2.4.7 ELISA was used to detect IL-4, IL-6, TNF-α and MMP-9

The cocultured cells were lysed and centrifuged, the supernatant was retrieved, and the contents of the related indexes were determined according to the requirements of the kit.

#### 2.4.8 Western blotting was used to detect the expression of the CCL2 and CCR2 protein

The collected and treated cells were added to the lysate and centrifuged at 12,000 r/min for 15 min, and the protein was quantified using the BCA method. Protein samples were transferred to the PVDF membrane after SDS-PAGE gel electrophoresis at room temperature for 2 h. Next, membranes were incubated with the primary antibody CCL2 and CCR2 (1:1,000) and incubated at 4°C overnight. The secondary antibody (1:10,000) was added, and incubated at room temperature for 2 h. Membranes were washed with PBST three times, and the ECL developer was added to visualize protein bands. Imaging was captured and analyzed by ImageJ software.

#### 2.4.9 RT-qPCR detected CCL2, CCR2 gene expression

The treated cells were collected, total RNA was extracted according to the instructions of the Trizol kit, and cDNA was synthesized by reverse transcription. The sequences of each quantitative PCR primer are shown in Table 1. The amplification mix volume was 20 μL: 2×SYBR Green qPCR Master Mix 10 μL, forward Primer (10 μM) 0.4 μL, Reverse Primer (10 μM) 0.4 μL, cDNA 3 μL, and RNase-free water 6.2 μL. The reaction conditions were as follows: 95°C 30 s, 95°C 15 s, and 60°C 30 s for 40 cycles. After the amplification reaction, the relative expression level of mRNA in each group was compared using the 2^−ΔΔCt^ method.

**TABLE 1:** Primer sequences.

Gene	Amplicon Size (bp)	Forward primer (5'→3′)	Reverse primer (5'→3′)
Hu-β-actin	96	CCC​TGG​AGA​AGA​GCT​ACG​AG	GGA​AGG​AAG​GCT​GGA​AGA​GT
Hu-CCL2	127	CAG​CAG​CAA​GTG​TCC​CAA​AG	CGG​AGT​TTG​GGT​TTG​CTT​GT
Hu-CCR2	110	AGT​CAA​CTG​GAC​CAA​GCC​AC	TGA​AAA​AGG​CTT​CTG​AAC​TTC​TCC

### 2.5 Protective effect of THSWD on brain injury in MCAO/R model rats

#### 2.5.1 Grouping and administration

Rats with successful modeling were randomly divided into 6 groups with 18 rats in each group, which were treated as follows: H-THSWD (18 g/kg), M-THSWD (9 g/kg), L-THSWD (4.5 g/kg), nimodipine (NMDP) (20 mg/kg), sham, and MCAO/R groups, respectively. The drug treatment group was treated by gavage according to the proportion of body weight 2 days after surgery, and the sham group and the MCAO/R group received the same amount of normal saline once a day for 7 consecutive days.

#### 2.5.2 Berderson score

After 7 days of administration, the neurological defects of the rats were scored, detailed scoring rules are shown in Table 2. Higher-scoring rats indicated more severe neurological deficits.

**TABLE 2:** Neurological function score.

Score	Symptoms seen in rats
0	Moved normally
1	The left front paw could not be successfully extended
2	Turned to the left when crawling
3	The rat’s body was skewed to the left when crawling
4	Unconscious, unable to crawl

#### 2.5.3 TTC staining was used to calculate the volume of cerebral infarction

One hour after the last administration, the rats were anesthetized, blood was collected from the abdominal aorta, followed by decapitation to harvest the brain, and frozen at −20°C for about half an hour. Specimens were cut into five coronal sections with a thickness of approximately 2 mm. The sections were then submerged in a 2% TTC solution, shielded from light by tinfoil, and incubated at 37°C for 30 min. Normal brain tissue was pink and the infarct area was white. After staining, tissue specimens were fixed in 4% paraformaldehyde, photographed, and recorded 24 h later. ImageJ was used for analysis and the infarct volume was calculated according to the formula.

$$\text{Corrected infarct volume }\left(\%\right)=\left\{\left[\text{total lesion volume}‐\left(\text{left hemisphere volume}‐\text{right hemisphere volume}\right)\right]/\text{right hemisphere volume}\right\}\times 100\%$$

#### 2.5.4 Hematoxylin and Eosin staining

The brain tissue was soaked in 4% paraformaldehyde solution overnight, dehydrated, embedded in paraffin, then sliced by an ultrathin microtome with a thickness of 5–8 μm, dried and stained with Hematoxylin and Eosin staining (HE), and observed under the microscope.

#### 2.5.5 ELISA was used to detect serum levels of IL-4, IL-6, TNF-α, and MMP-9

Rat abdominal aorta blood was collected, left at room temperature for 30 min, then placed in a 4°C centrifuge, and centrifuged at 3,000 r/min for 10 min. The upper serum layer was retrieved according to the kit requirements to determine the content of relevant indicators.

#### 2.5.6 Immunohistochemistry for the expression of the claudin-5, CD86 (M1), and CD206 (M2) protein in the cerebral cortex

After embedding in paraffin, brain tissue was cut and immunohistochemical staining was performed. Monoclonal antibodies for claudin-5, CD86, and CD206 were diluted 1:500, respectively. The distribution of target protein was observed under the microscope and the brown particles showed positive expression. ImageJ software measured the optical density (OD) values of brown particles on each wall of the wall of the blood vessel under each visual field and calculated the average value, representing the expression of the corresponding protein.

#### 2.5.7 Immunofluorescence detected CCR2^+^/CD68^+^ macrophages

Paraffin specimens of brain tissue were prepared and sectioned. The sections were blocked in 3% hydrogen peroxide at room temperature for 30 min, and then rinsed in distilled water for 3 min. After sealing with 10% goat serum, take 1 μL of the original solutions of CCR2 and CD68 antibodies, prepare the working antibody solution, add the primary antibody, and incubate at 4°C overnight. The next day, the slices were rinsed with TBST, soaked several times, added with secondary antibody, incubated and removed from the oven. After removing TBST, each section was exposed to 50 μL DAPI, incubated in the dark, and rinsed with TBST. The slides were sealed with fluorescent sealer and examined under the microscope and then stored away from light. Pathological changes were observed by fluorescence microscopy. Immunofluorescence staining was quantified using ImageJ.

#### 2.5.8 Western blotting detected the expression of the CCL2 and CCR2 proteins in brain tissue

Brain tissue was lysed on ice for 40 min, centrifuged, and protein was extracted and Western blotting followed the procedure outlined in Section 2.4.8.

#### 2.5.9 RT-qPCR detection of CCL2 and CCR2 protein expression in brain tissue

Brain tissue was used to prepare a homogenate. RT-PCR was performed as described in Section 2.4.9.

### 2.6 Statistical analysis

The experimental data were statistically analyzed and plotted using GraphPadPrism v.8.0.2 software, and the results were expressed as x ± s. The independent *t*-test was used for comparison between the two groups. *p* < 0.05 was considered statistically significant.

## 3 Results

### 3.1 Potential immunoinflammatory-related targets of THSWD in the treatment of ischemic stroke

#### 3.1.1 Differential gene expression and GSEA analysis following THSWD treatment for ischemic stroke

**FIGURE 1:**
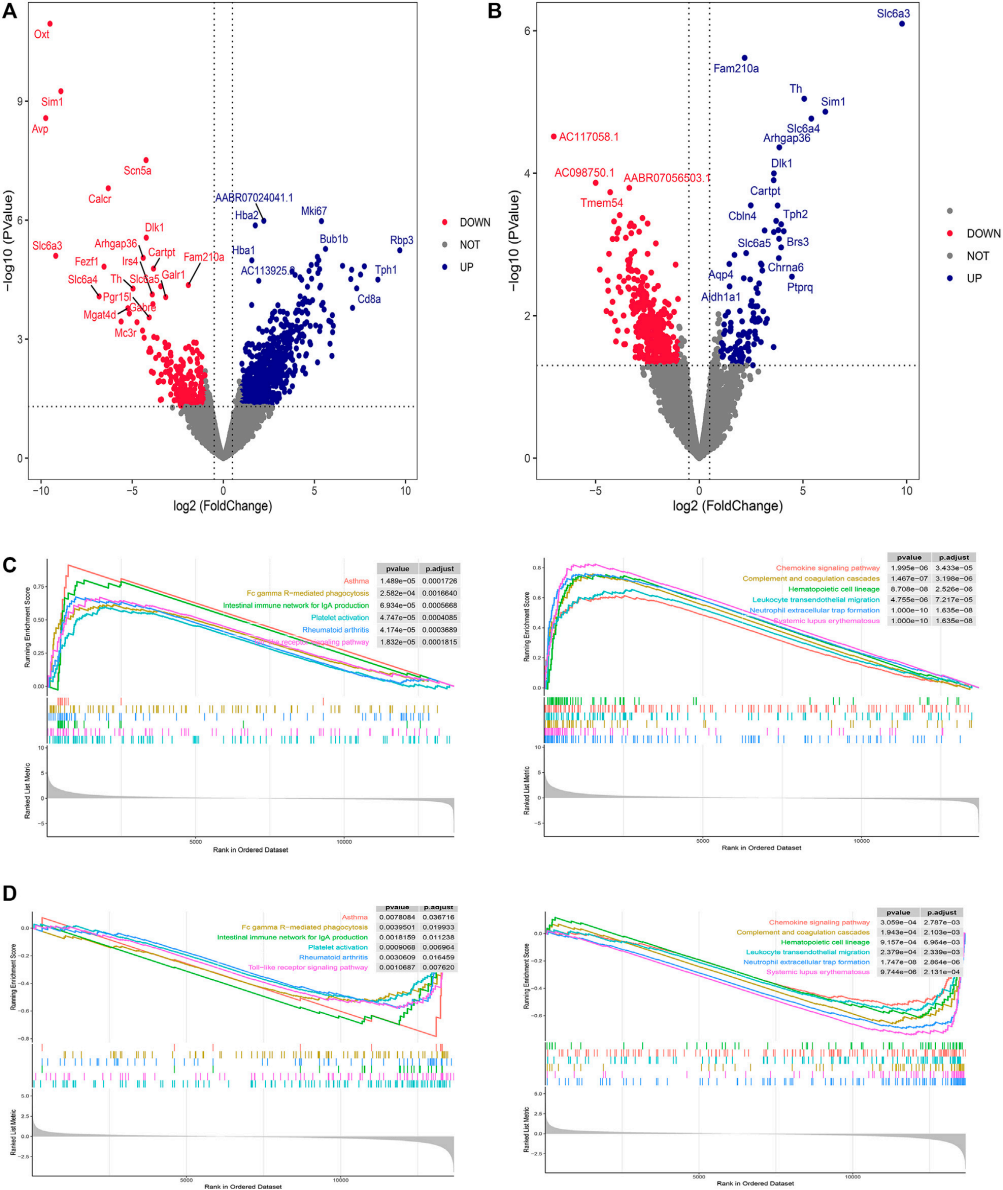
Differential gene screening and GSEA analysis of THSWD treatment for ischemic stroke. **(A)** MVSC volcano map, **(B)** DVSM volcano map, **(C, D)** Gene set enrichment analysis of mRNA transcriptome sequencing data.

DEG screening for DVSM and MVSC was performed using R.4.2.1, A *P*-value <0.05 and the absolute value of logFC >1 were set as intermediate values. In total, 806 upregulated genes and 226 downregulated genes were detected following MVSC treatment (Figure 1A), and 401 downregulated genes and 114 upregulated genes were identified for DVSM, for a total of 515 DEGs. Of these, THSWD downregulated 285 genes and upregulated 61 genes (Figure 1B). The expression profile of the comparison group DVSM and MVSC was analyzed by GSEA, *P*-valueCutoff = 0.05. MVSC activated 24 immune-related pathways (Figure 1C) and DVSM activated 12 immune-related pathways (Figure 1D) and immunoinflammatory pathways were significantly enriched.

#### 3.1.2 Screening and enrichment analysis of immunoinflammatory genes following treatment with THSWD for ischemic stroke

**FIGURE 2:**
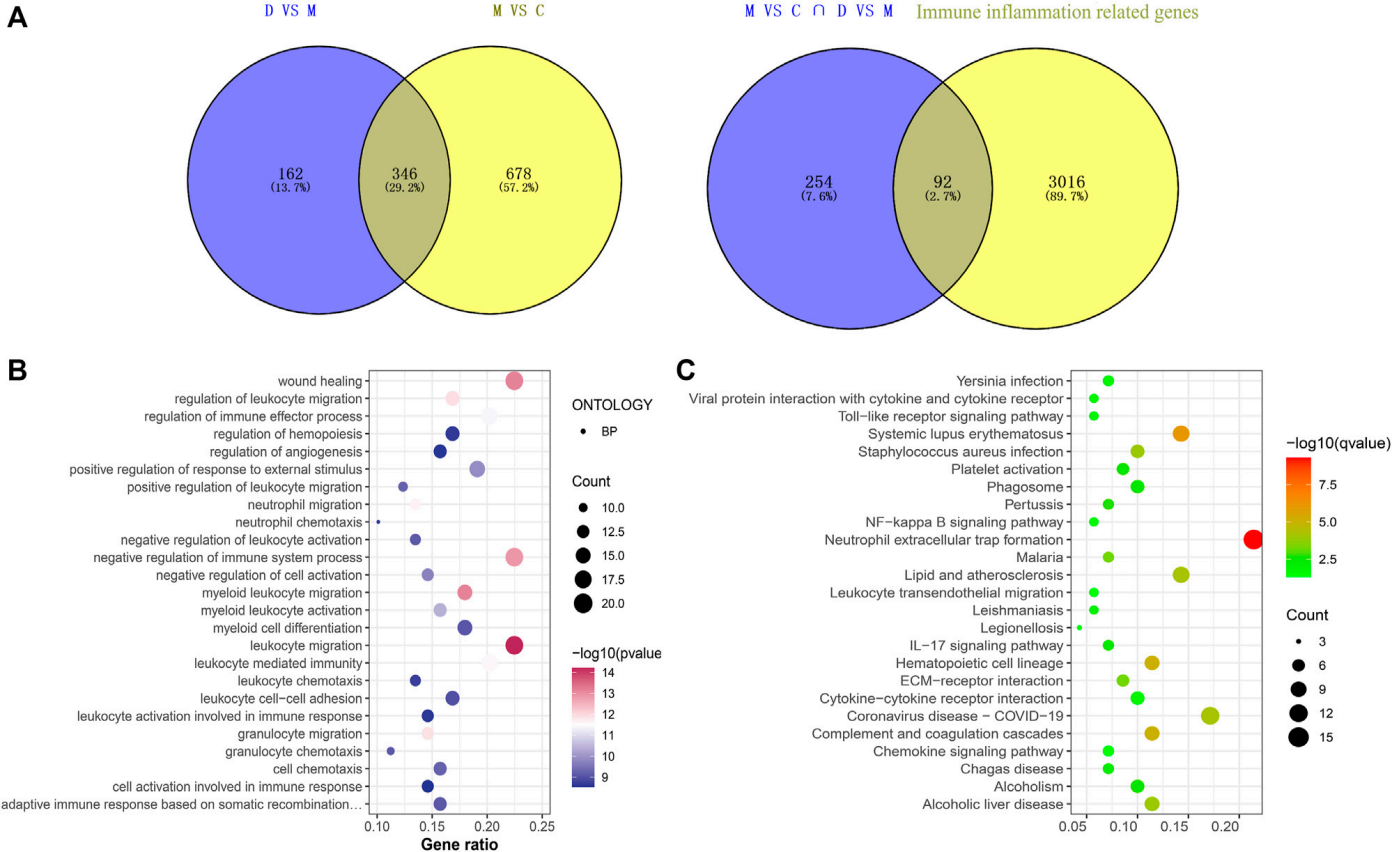
**(A)** Immune inflammation-related gene screening **(B)** Immunoinflammatory-related differential gene GO enrichment analysis (Top25). **(C)** Immunoinflammatory-related differential gene KEGG analysis (Top25).

There were 92 immunoinflammatory-related differential genes in the THSWD intervention for stroke (Figure 2A). The GO enrichment analysis of immunoinflammation-related differential genes is shown in Figure 2B and the KEGG enrichment analysis is shown in Figure 2C.

#### 3.1.3 PPI network construction and screening of key genes

**FIGURE 3:**
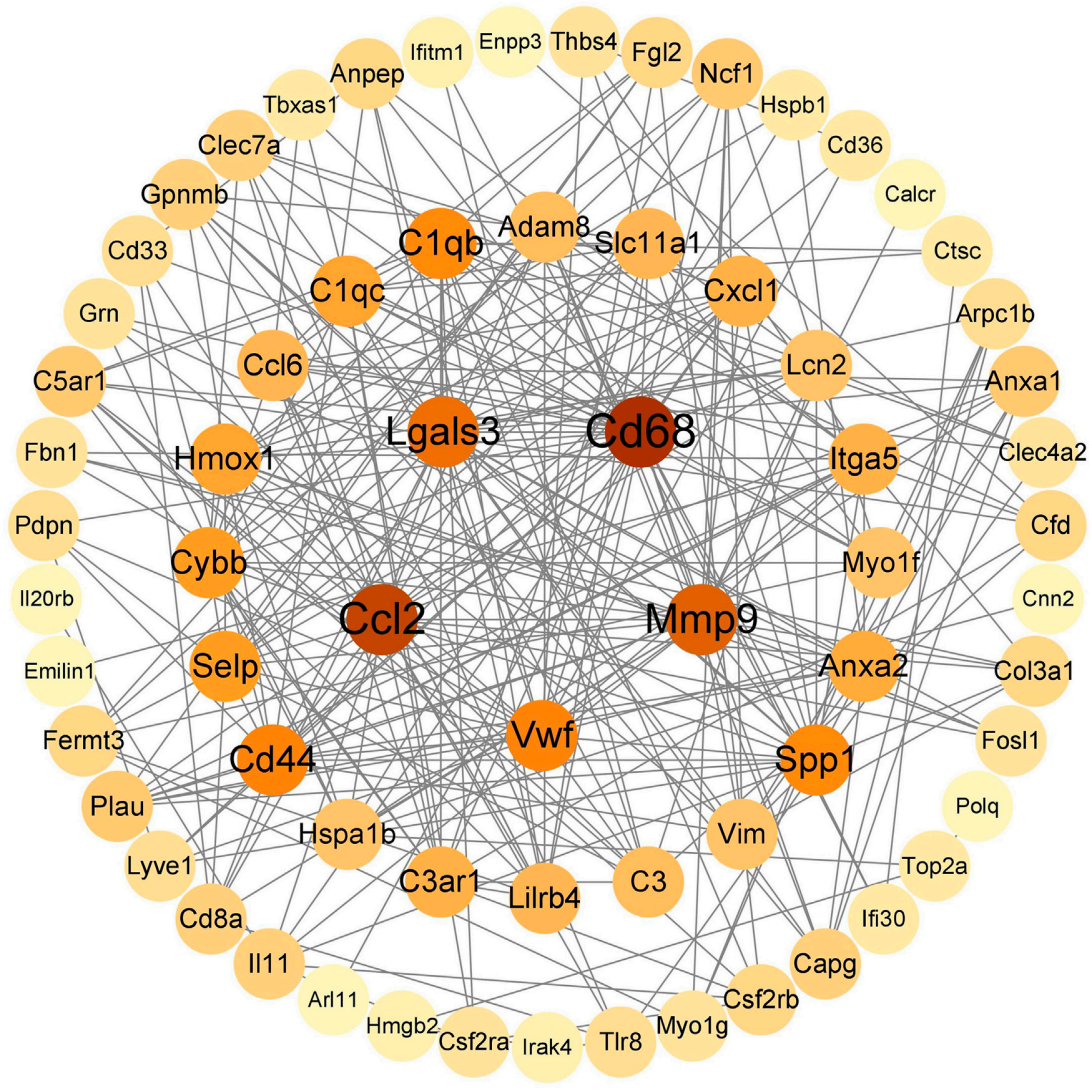
HUB gene screening.

String (https://cn.string-db.org/) was used to construct a PPI network map of 92 immunoinflammatory genes, which was imported into Cytoscape v.3.9.1 to visualize the selected genes with the MCC algorithm. The five genes with the highest scores (*Cd68, Ccl2, Mmp9, Lgals3, Cd44*) were identified as key genes (Figure 3).

### 3.2 THSWD alleviated OGD/R-induced HBMEC damage by inhibiting the CCL2/CCR2 axis

#### 3.2.1 Inhibition of the CCL2/CCR2 axis protected HBMEC cells from OGD/R-induced damage

**FIGURE 4:**
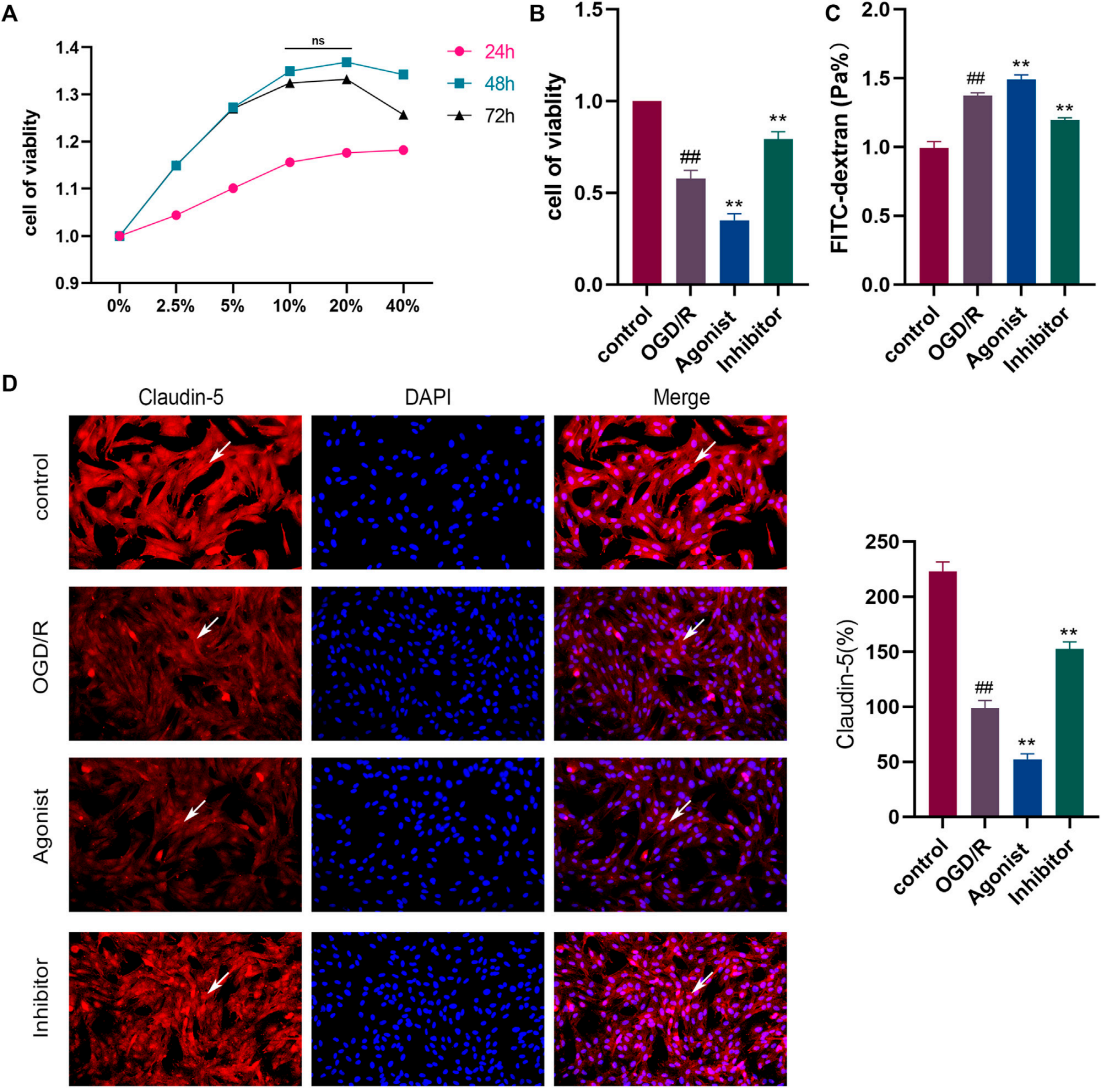
Inhibition of the CCL2/CCR2 axis can attenuate OGD/R damage in HBMEC. **(A)** Screening of drug-containing serum. **(B)** Effect of the CCL2/CCR2 axis on HBMEC activity. **(C)** FITC permeability of HBMEC. **(D)** Effect of the CCL2/CCR2 axis on claudin-5. ^##^ *P* < 0.01 vs. control; ^**^ *p* < 0.01 vs. OGD/R.

The results of the CCK-8 assay found that the strongest cell proliferation effect was observed in the drug-containing serum containing the 10% concentration and 48 h after administration, which was then selected as the conditions for subsequent experiments (Figure 4A). CCL2/CCR2 axis inhibitors antagonized OGD/R-induced HBMEC damage, and decreased cell activity was observed following exposure to OGD/R. CCR2 inhibitors increased cell viability (*p* < 0.01), whereas CCL2/CCR2 axis agonists induced the opposite effect (*p* < 0.01) (Figure 4B). The permeability of HBMEC was detected by FITC-dextran. OGD/R injury significantly improved the permeability of HBMEC cells (*p* < 0.01), whereas the CCL2/CCR2 axis inhibitor significantly reversed this increase (*p* < 0.01) (Figure 4C). The expression of claudin-5 following OGD/R injury was reduced compared to the control group (*p* < 0.01), indicating that HBMEC was damaged, which was reversed by treatment with CCL2/CCR2 axis inhibitors (*p* < 0.01), indicating that CCL2/CCR2 axis inhibitors maintained endothelial cell function (Figure 4D).

#### 3.2.2 Inhibition of the CCL2/CCR2 axis reduced macrophage polarization and the release of inflammatory factors

**FIGURE 5:**
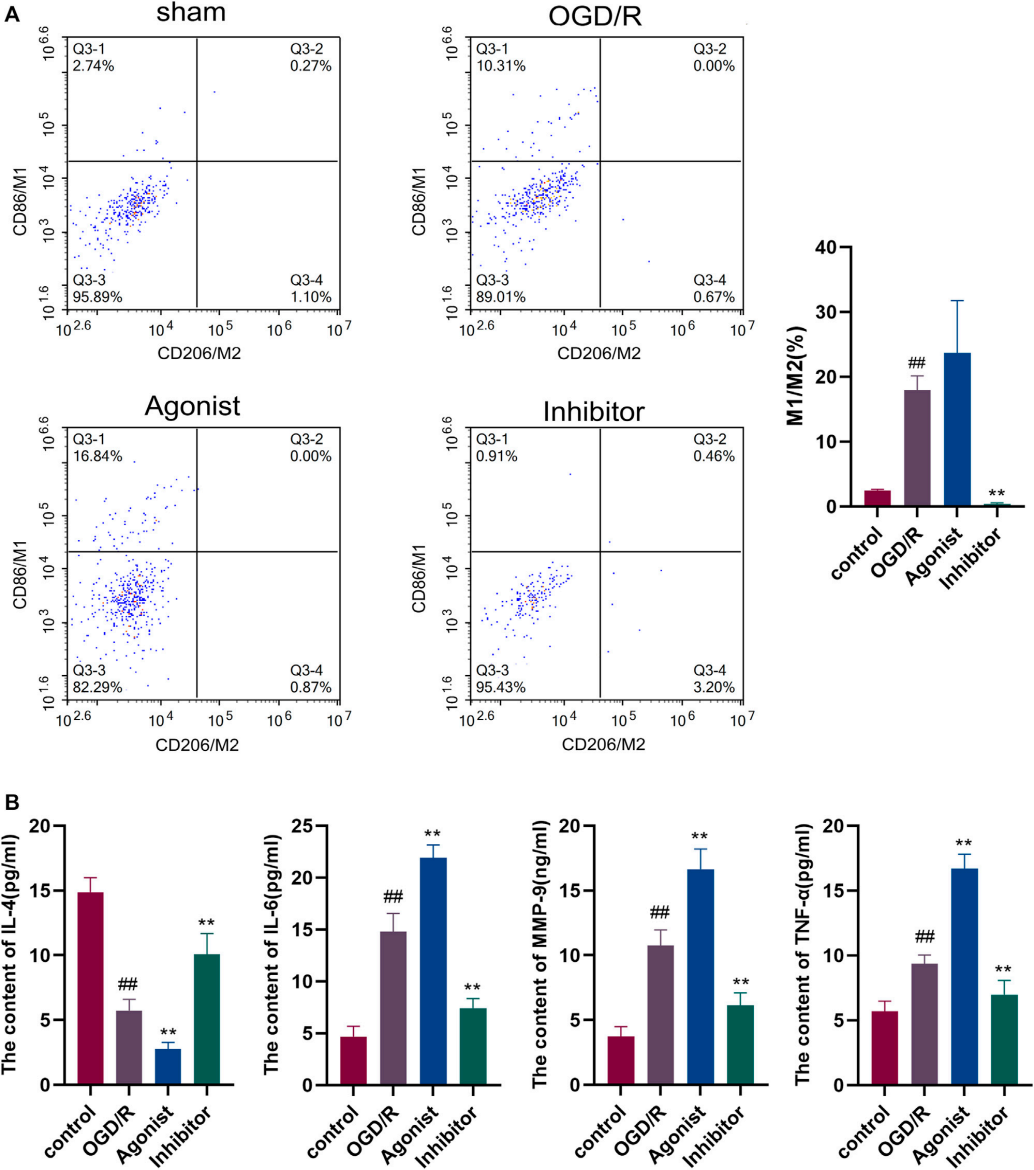
Inhibition of the CCL2/CCR2 axis can reduce macrophage polarization and inflammatory factor release. **(A)** Macrophage polarization. **(B)** Inflammatory factor. ^##^ *p* < 0.01 vs. control; ^**^ *p* < 0.01 vs. OGD/R.

Flow cytometry was used to detect macrophage polarization, and it was found that the CCL2/CCR2 axis inhibitors reduced M1/M2 (*P*<0.01), indicating that the CCL2/CCR2 axis inhibitors caused macrophages to switch from the pro-inflammatory subgroup (M1) to the anti-inflammatory subgroup (M2) (Figure 5A). ELISA was used to evaluate the release of inflammatory cytokines in all experimental groups. The expression of pro-inflammatory cytokines IL-6, MMP-9, and TNF-а were decreased in the CCL2/CCR2 axis inhibitor group (*p* < 0.01). In contrast, the expression of anti-inflammatory factor IL-4 was significantly increased (*p* < 0.01) (Figure 5B).

#### 3.2.3 Expression of proteins and genes related to the CCL2/CCR2 axis

**FIGURE 6:**
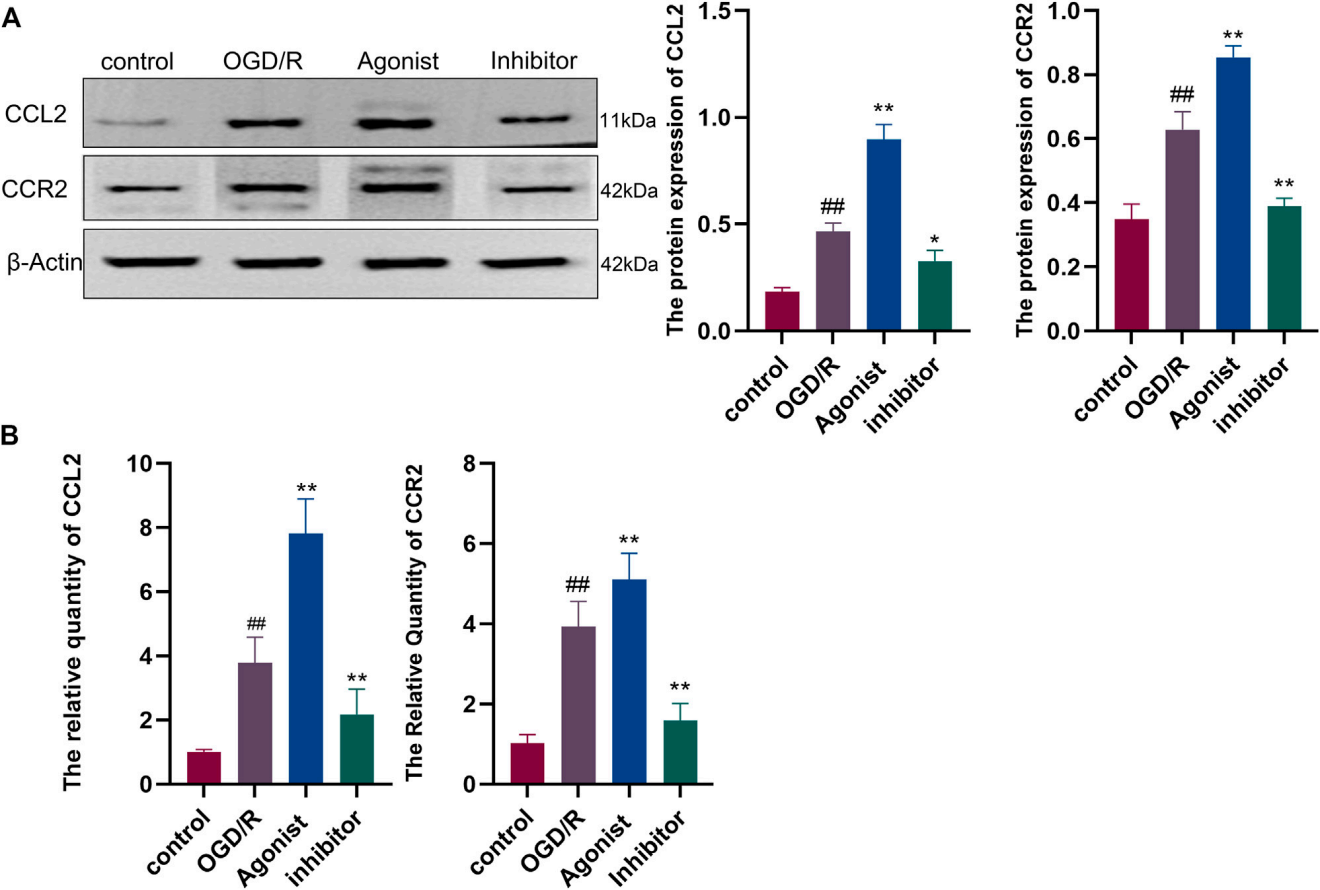
Expression of proteins and genes associated with the CCL2/CCR2 axis. **(A)** Western blotting; **(B)** RT-qPCR. ^##^ *p* < 0.01 vs. control; ^*^ *p* < 0.05, ^**^ *p* < 0.01 vs. OGD/R.

Western blotting showed that CCL2/CCR2 axis inhibitor significantly inhibited the expression of CCL2 and CCR2 compared with other groups (*p* < 0.01, *p* < 0.05) (Figure 6A), and RT-qPCR indicated that CCL2 and CCR2 expression was downregulated in the CCL2/CCR2 axis inhibitor group (*p* < 0.01) (Figure 6B).

#### 3.2.4 THSWD alleviated the damage to the OGD/R model of HBMEC

**FIGURE 7:**
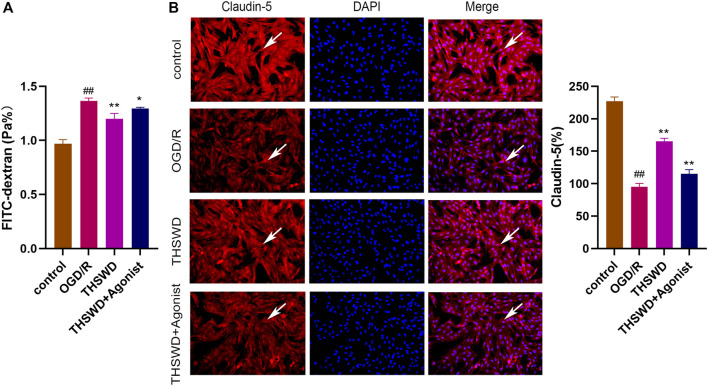
THSWD inhibition of the CCL2/CCR2 axis alleviates HBMEC cell injury. **(A)** FITC permeability of HBMEC. **(B)** THSWD inhibits the effect of the CCL2/CCR2 axis on claudin-5 expression. ^#^ *P* < 0.05, ^##^ *P* < 0.01 vs. control; ^*^ *p* < 0.05, ^**^ *p* < 0.01 vs. OGD/R.

The FITC glucan assay showed that the THSWD group alleviated HBMEC permeability (*p* < 0.01) and THSWD could reverse the increased permeability of HBMEC induced by CCL2/CCR2 axis agonists (*p* < 0.05) (Figure 7A). Furthermore, the immunofluorescence results revealed that claudin-5 expression was increased in the THSWD group (*p* < 0.01), and that THSWD could reverse the damage to HBMEC caused by CCL2/CCR2 axis agonists (*p* < 0.01) (Figure 7B).

#### 3.2.5 THSWD inhibited macrophage polarization

**FIGURE 8:**
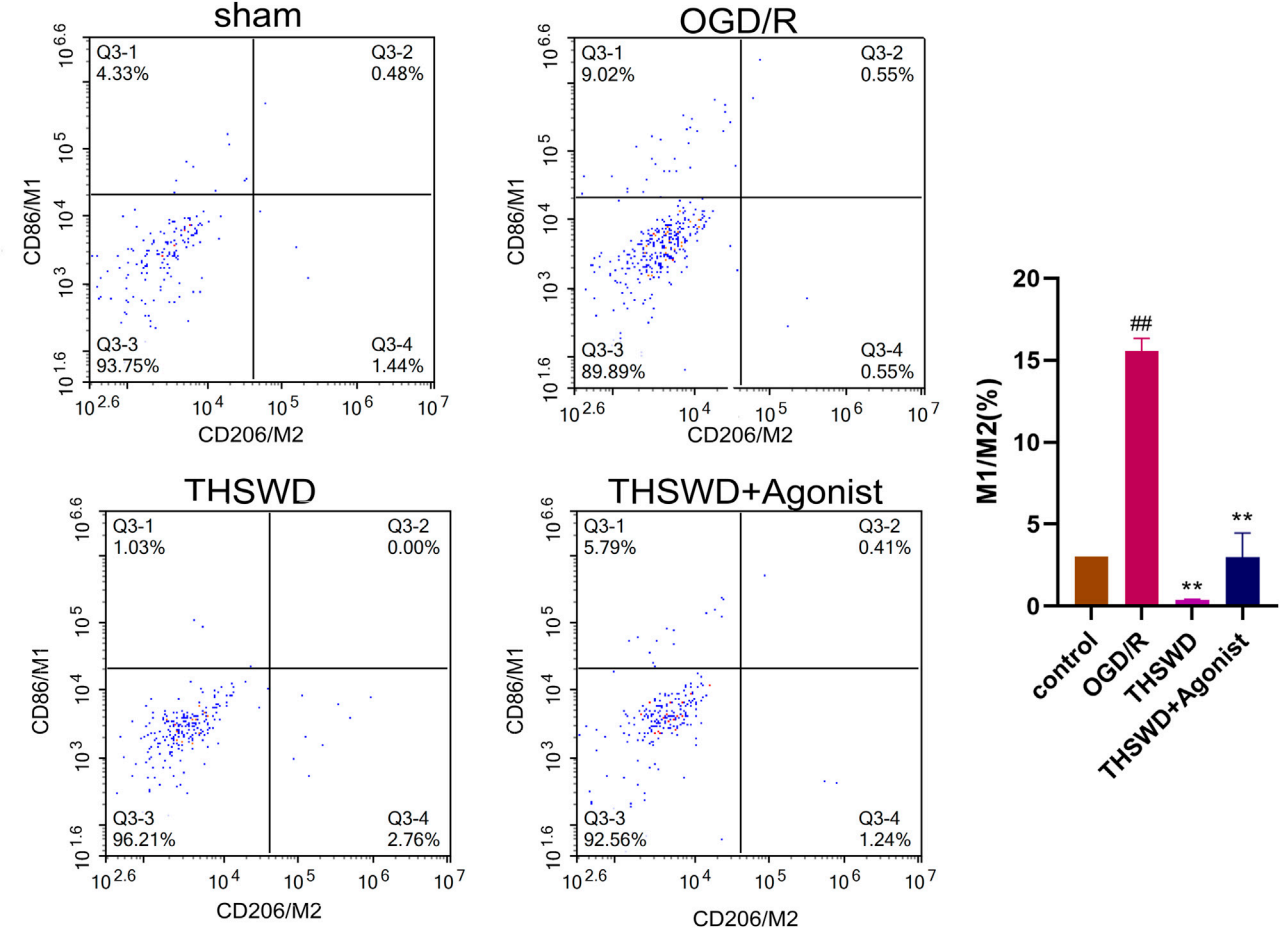
THSWD inhibits the CCL2/CCR2 axis and inhibits macrophage polarization. ^##^ *P* < 0.01 vs. control; ^**^ *p* < 0.01 vs. OGD/R.

After co-culture of endothelial cells and macrophages, flow cytometry showed that the proportion of macrophages M1 and M2 (M1/M2) in the THSWD group was reduced (*p* < 0.01). THSWD also reversed macrophage-induced polarization in the CCL2/CCR2 axis agonist group (*p* < 0.01) (Figure 8).

### 3.3 THSWD exerted a protective effect on MCAO/R rats by inhibiting the CCL2/CCR2 axis

#### 3.3.1 THSWD alleviated nerve function injury and blood-brain barrier injury in MCAO/R rats

**FIGURE 9:**
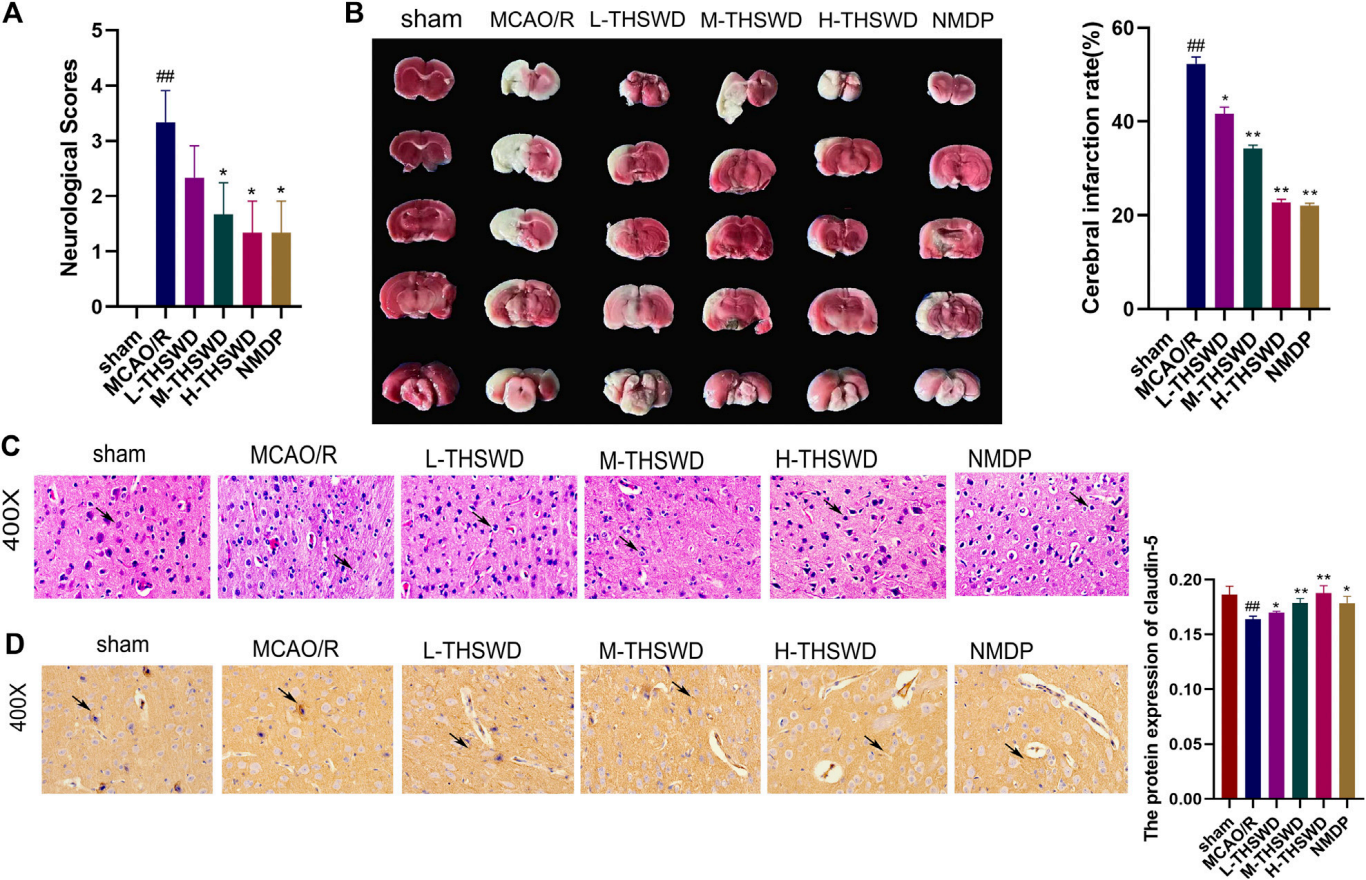
**(A)** THSWD decreased the neurological function score of MCAO/R rats (n = 10); **(B)** TTC staining showed cerebral infarction volume (n = 3); **(C)** neuronal damage (HE, ×400); **(D)** THSWD increased the expression of claudin-5 in MCAO/R rats. ^#^ *P* < 0.05, ^##^ *P* < 0.01 vs. sham; ^*^ *p* < 0.05, ^**^ *p* < 0.01 vs. MCAO/R.

Compared with the sham group, the neural function score of rats in the MCAO/R group was increased (*p* < 0.01) (Figure 9A); TTC staining showed infarction on the same side of the brain section (*p* < 0.01) (Figure 9B). HE staining revealed disordered neuronal cell arrangement, damaged cell structure, and atrophy of nucleus in the brain tissue of the MCAO/R group (Figure 9C). Rats in the THSWD group and the NMDP group showed a decrease in the nerve function score (*p* < 0.05), a decrease in the area of cerebral infarction area (*p* < 0.05, *p* < 0.01), and an improvement in HE-stained neurons damage, which was dose dependent. On immunohistochemistry, the expression of the claudin-5 protein in the MCAO/R group was lower than in the sham group (*p* < 0.01), whereas the expression of the claudin-5 protein in the H-THSWD, M-THSWD, L-THSWD, and NMDP groups increased (*p* < 0.05, *p* < 0.01) (Figure 9D).

#### 3.3.2 THSWD inhibited infiltration and polarization of brain macrophages in MCAO/R rats

**FIGURE 10:**
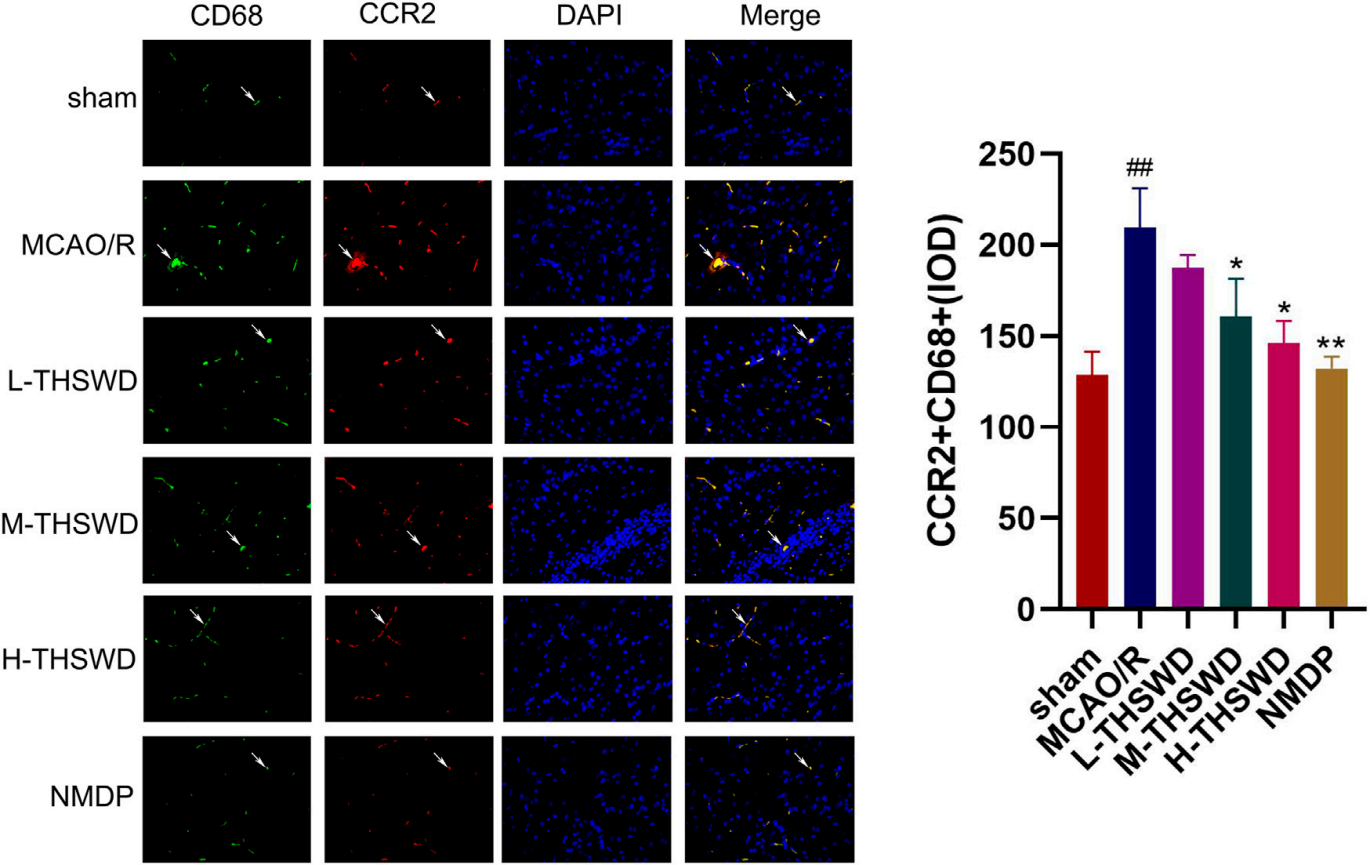
The intensity of CCR2^+^/CD68^+^cell reaction; ^##^ *P* < 0.01 vs. sham; ^*^ *p* < 0.05, ^**^ *p* < 0.01 vs. MCAO/R.

**FIGURE 11:**
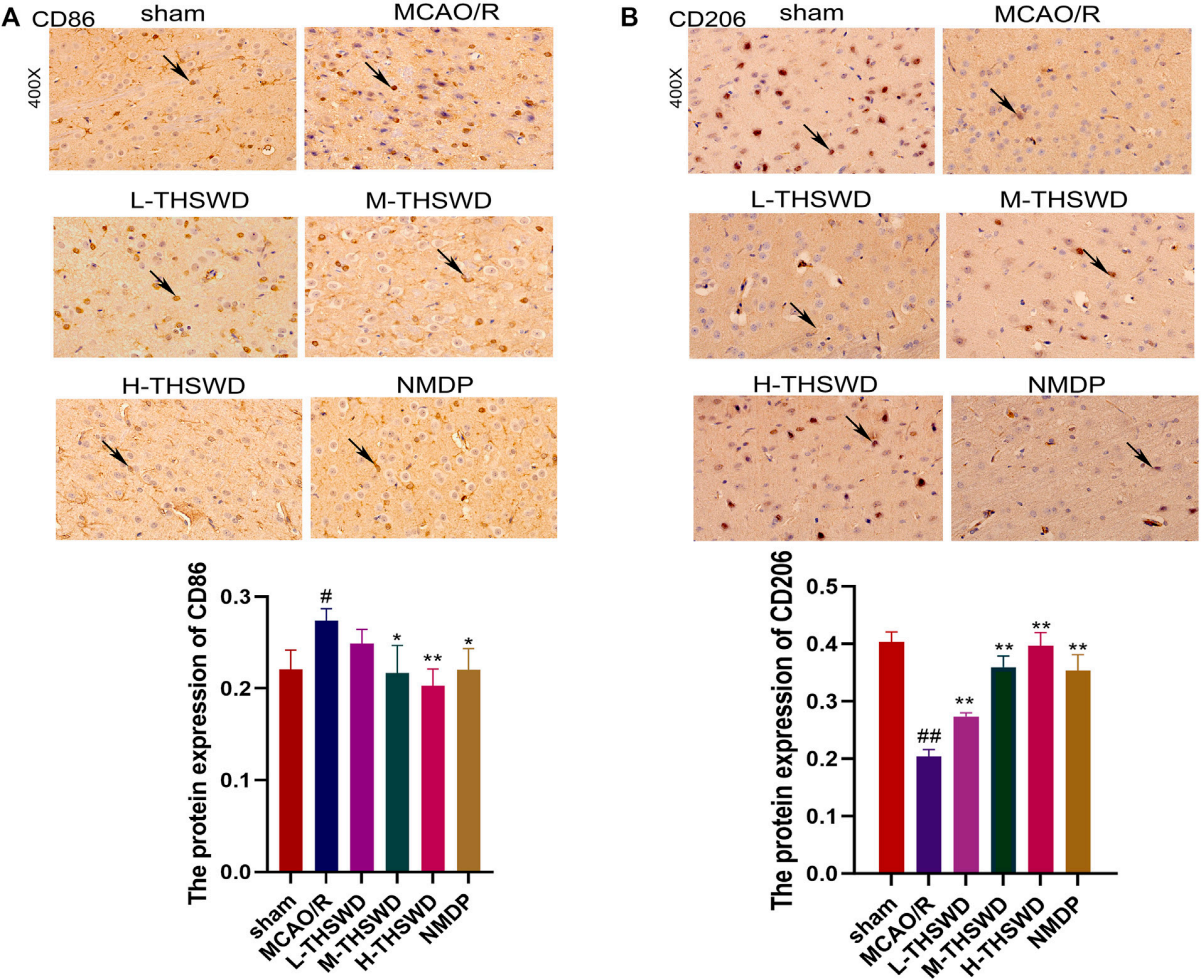
Expression of CD86 (M1) and CD206 (M2). **(A)**: Expression of CD86 (M1); **(B)**: Expression of CD206 (M2). ^#^ *P* < 0.05, ^##^ *P* < 0.01 vs. sham; ^*^ *p* < 0.05, ^**^ *p* < 0.01 vs. MCAO/R.

It is well known that chemokine-chemokine receptor interactions induce the recruitment of inflammatory cells, and our results showed that most inflammatory cells expressing CCR2 were CD68-positive cells, indicating that the CCL2/CCR2 axis was significantly correlated with increased macrophage recruitment. Compared to the sham group, the reaction intensity of CCR2^+^/CD68^+^ cells in the MCAO/R group significantly increased (*p* < 0.01), while this phenomenon was reversed in the H-THSWD, M-THSWD, and NMDP groups (*p* < 0.05, *p* < 0.01) (Figure 10). Immunohistochemical identification of CD86 (M1) and CD206 (M2) macrophages showed that compared to the sham group, the number of M1 macrophages increased (*p* < 0.05) and M2 macrophages decreased significantly (*p* < 0.01) in the MCAO/R group (Figures 11A, B). The H-THSWD, M-THSWD, L-THSWD, and NMDP groups all reversed the above phenomenon. (*p* < 0.05, *p* < 0.01).

#### 3.3.3 THSWD inhibited the release of inflammatory factors and the expression of genes and proteins of the CCL2/CCR2 axis in the hippocampus of MCAO/R rats

**FIGURE 12:**
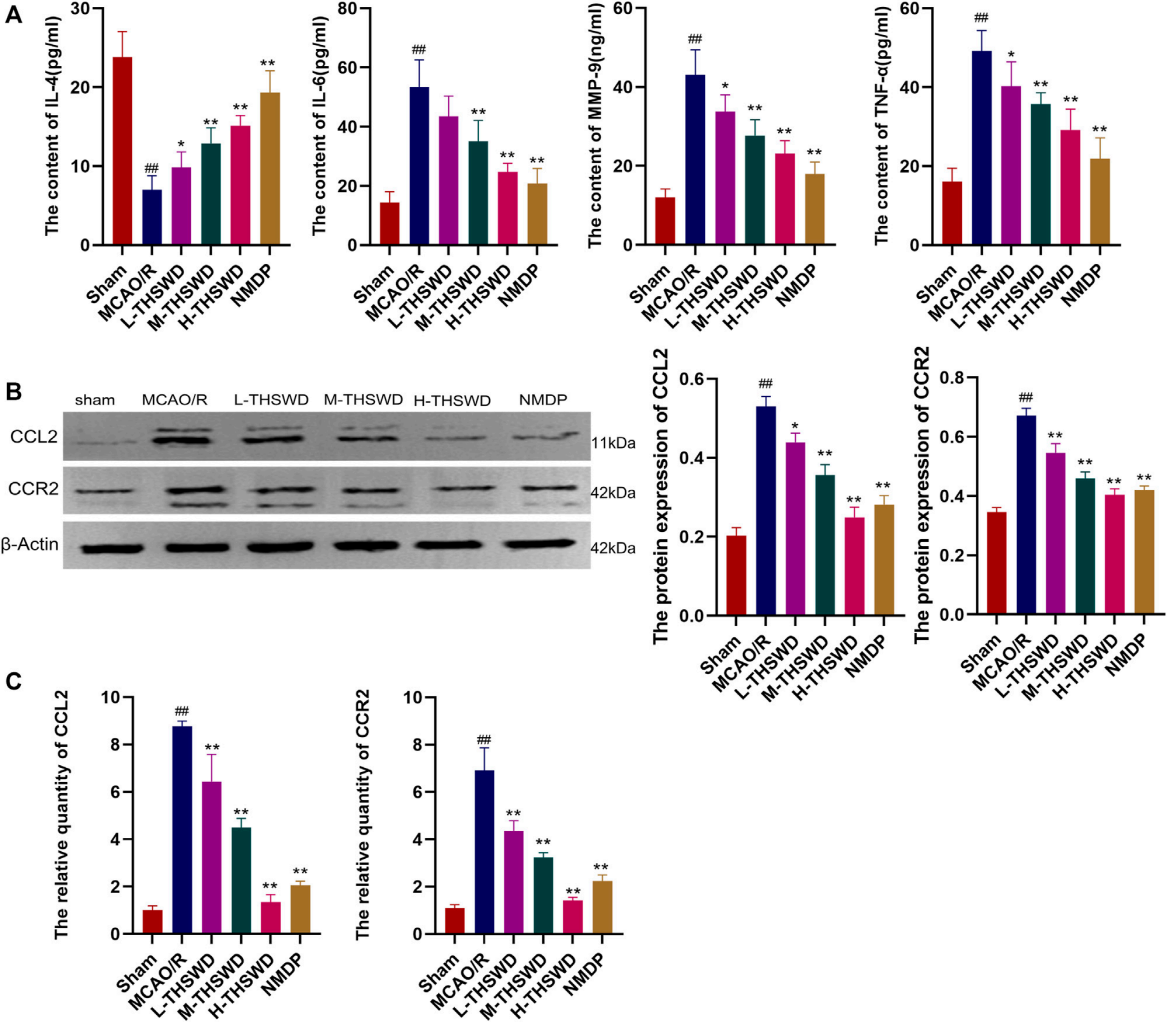
THSWD inhibits the release of inflammatory factors and the CCL2/CCR2 axis. **(A)** Inflammatory cytokines. **(B)** Western blotting. **(C)** RT-qPCR. ^#^ *P* < 0.05, ^##^ *P* < 0.01 vs. sham; ^*^ *p* < 0.05, ^**^ *p* < 0.01 vs. MCAO/R.

The ELISA showed that the release of proinflammatory factors MMP-9, TNF-α and IL - 6 by the MCAO/R group were significantly higher than those of the sham group (*p* < 0.01), IL-4 showed the opposite trend (*p* < 0.01), whereas THSWD and NMDP treatment groups reversed the expression of inflammatory factors (*p* < 0.05, *p* < 0.01) (Figure 12A); In addition, the gene and protein expression of CCL2 and CCR2 in the MCAO/R group was higher than that in the sham group (*p* < 0.01) (Figures 12B, C). Similarly, this phenomenon was reversed in the THSWD and NMDP groups (*p* < 0.05, *p* < 0.01).

## 4 Discussion

Bioinformatics has been extensively used in the study of ischemic stroke. Li et al. (2020) using bioinformatics, found that the expression of CCL2 in the ischemic stroke group increased and promoted the progression of ischemic stroke by activating chemokine signaling and other pathways. In this study, through downstream analysis of transcriptome data from MCAO/R rats treated with THSWD, GSEA showed that gene sets were significantly enriched in the immunoinflammatory pathway and key genes such as Cd68 and Ccl2 were finally selected.

Macrophages are highly plastic, and when stimulated by the microenvironment, their polarization is triggered; they are usually divided into M1 and M2 types according to their cell function and metabolic pattern (Deng et al., 2023), M1 macrophages initiate pro-inflammatory responses, whereas M2 macrophages mediate anti-inflammatory processes. An imbalance in the M1-M2 polarization of macrophages is often associated with various diseases or inflammation (Wang et al., 2014). The phenotypic polarization of macrophages is usually studied by detecting M1-polarized macrophage markers CD86, CD16, and iNOS and M2-polarized macrophage markers CD206, Arg, and Ym1 (Spiller et al., 2014). BBB integrity is damaged after ischemic stroke (Ahmad et al., 2019). Natural immune cells are activated to migrate to the ischemic site in response to chemokines and pass through the damaged BBB (Zeng et al., 2022). Subsequently, macrophages in ischemic brain tissue are activated to further release inflammatory factors, exacerbating the chemotaxis of innate immune cells (Bayraktutan, 2019). Macrophages are one of the main drivers of inflammation during ischemia and stroke, and it has been shown that SP (undeceptide) is highly effective in the early control of ischemic stroke-induced inflammation. Possibly by recruiting M2 monocytes/macrophages into the injured brain, the M2 microglia/macrophages in the rat brain hemispheres after SP injection were significantly increased and inflammation was reduced. Thus, SP treatment significantly inhibited the destructive inflammatory response and protected and repaired the tissue microenvironment (Ahn et al., 2023). In this study, CD86 and CD206 were detected at the cellular and animal levels, respectively, and THSWD treatment resulted in a significant decrease in M1 macrophages and a significant increase in M2 macrophages compared with the model group. It indicated that THSWD could inhibit macrophage polarization.

**FIGURE 13:**
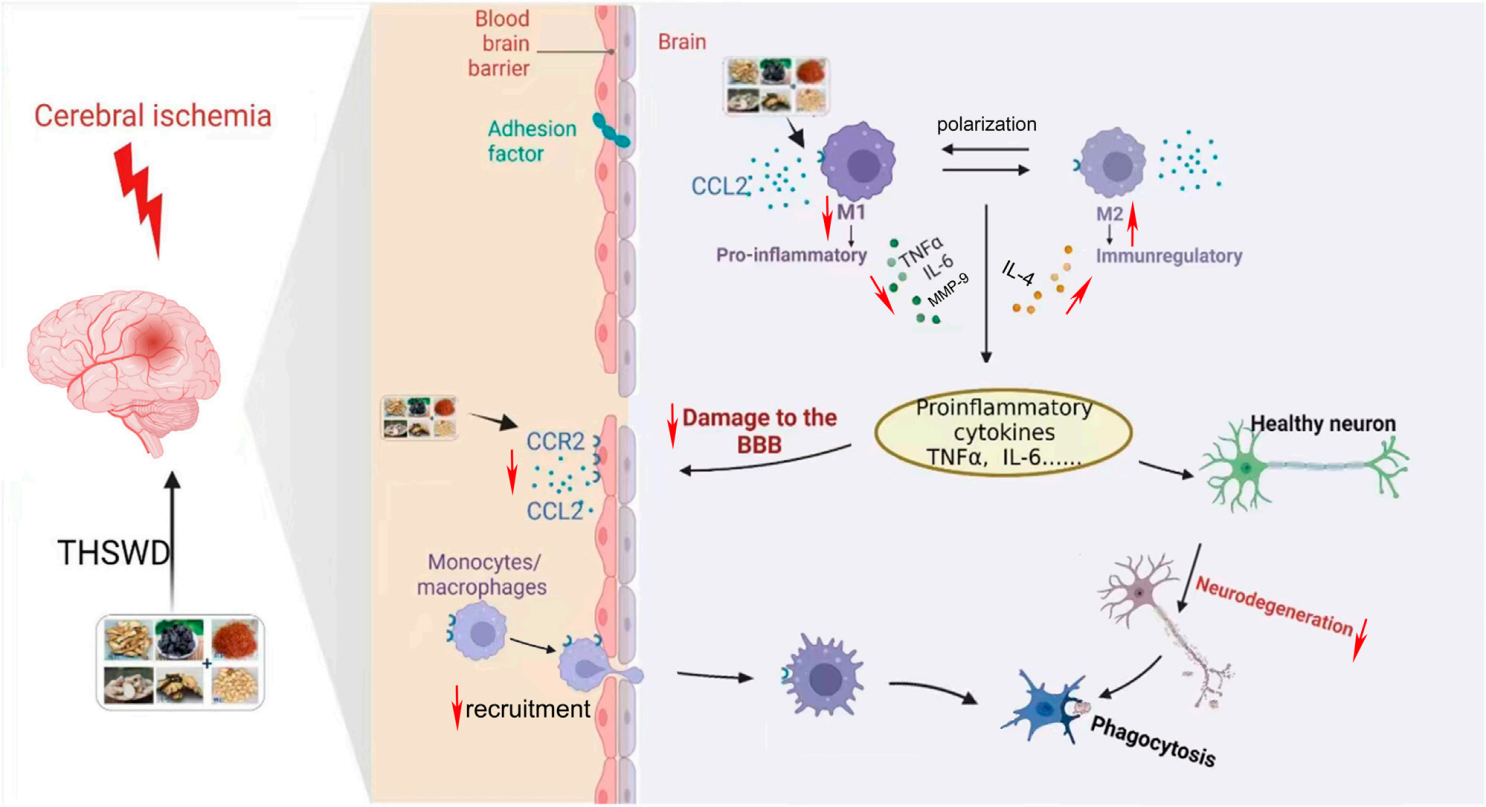
Mechanisms of CCL2/CCR2 axis in ischemic stroke and intervention of THSWD.

CCR2 is a major macrophage chemokine receptor, and together with its ligand CCL2, plays an important role in neuroinflammation, mediating the recruitment of infiltrating macrophages and resident microglia to inflammatory sites in the central nervous system (Bianconi et al., 2018). Furthermore, downregulation of CCL2/CCR2 can inhibit macrophage infiltration and reduce microglial/macrophage M1 polarization (Yang et al., 2020). Meanwhile, CCL2 has a destructive effect on the BBB, which is directly mediated by the expression of CCR2 in endothelial cells (Skelly et al., 2013) After the CCR2 gene is knocked out, the effect of CCL2 on the permeability of the BBB is eliminated (Geng et al., 2022). In conclusion, inhibition of CCL2/CCR2 can reduce cerebral ischemic injury through multiple mechanisms (Stowe et al., 2012; Xu et al., 2022). In this study, we found that inhibition of the CCL2/CCR2 axis led to a reduction in macrophage polarization and a decrease in the permeability of HBMEC. Conversely, activation of the CCL2/CCR2 axis resulted in opposite effects. Furthermore, THSWD significantly suppressed the release of inflammatory cytokines and the expression of CCL2/CCR2 axis genes and proteins in the hippocampus of MCAO/R rats. Additionally, THSWD was able to reverse the damage to HBMEC and the polarization of macrophages caused by an agonist of the CCL2/CCR2 axis. This indicates that THSWD can antagonize the effects induced by CCL2/CCR2 axis agonists, thereby inhibiting the polarization of M1 macrophages, reducing inflammatory damage, and protecting the BBB (Figure 13).

As previously mentioned, this study used bioinformatics to screen genes related to the immune inflammation of ischemic stroke treated with THSWD, conducted an in-depth study of the potential mechanisms by which THSWD regulates the CCL2/CCR2 axis, and provided a theoretical basis for the preclinical research of THSWD in the treatment of ischemic stroke. However, there are still areas that can be improved: 1) Conduct gene knockout studies on CCL2 to verify its impact on ischemic stroke and the effects of THSWD; 2) Carry out in-depth studies on other immune-inflammatory related genes (Mmp9, Lgals3, Cd44) identified through bioinformatics.

## 5 Conclusion

The CCL2/CCR2 axis is involved in the occurrence and development of ischemic stroke and may play a protective role against ischemic stroke injury in rats by reducing macrophage infiltration and inhibiting macrophage polarization of the M1 type, thus reducing inflammatory damage and protecting the blood-brain barrier. THSWD may play a protective role in inflammation after ischemic stroke by regulating the CCL2/CCR2 axis.

## Data Availability

The original contributions presented in the study are included in the article/supplementary material, further inquiries can be directed to the corresponding authors.
